# The impact of telephone-based telemedicine on unplanned hospital visits and mortality risk during the COVID-19 pandemic: a study from a middle-income country

**DOI:** 10.1186/s12877-025-06588-z

**Published:** 2025-12-02

**Authors:** Unchana Sura-amonrattana, Kasemsan Kertkiatkachorn, Supawadee Sainimnuan, Rinrada Preechitkul, Ponnapa Petchthai, Pattara Leelahavarong, Jeeranan Jantaraprapan, Sutisa Pitiyarn, Arunotai Siriussawakul, Varalak Srinonprasert, Chairat Permpikul

**Affiliations:** 1 Division of Geriatric Medicine, Department of Medicine, Faculty of Medicine Siriraj Hospital, Mahidol University, Bangkok, Thailand; 2 Department of Social Medicine, Prapokklao Hospital, Chanthaburi, Thailand; 3 Faculty of Medicine Siriraj Hospital, Siriraj Health Policy Unit, Mahidol University, Bangkok, Thailand; 4 Department of Nursing, Faculty of Medicine Siriraj Hospital, Mahidol University, Bangkok, Thailand; 5 Department of Anesthesiology, Faculty of Medicine Siriraj Hospital, Mahidol University, Bangkok, Thailand; 6 Research Department, Faculty of Medicine Siriraj Hospital, Siriraj Geriatric Internal Medicine Research Group, Mahidol University, Bangkok, Thailand; 7 Division of Critical Care, Department of Medicine, Faculty of Medicine Siriraj Hospital, Mahidol University, Bangkok, Thailand

**Keywords:** Telephone-based, Telemedicine, Unplanned visits, Older people, COVID-19 pandemic

## Abstract

**Background:**

Providing care via telemedicine was suggested worldwide during the COVID-19 pandemic. A new care model and service flow using telephone-based telemedicine (2T SAVE-COVID project) was established to provide care for patients at the Department of Medicine during the pandemic. This study aimed to investigate the clinical outcomes of patients after receiving care through telemedicine in the project.

**Methods:**

A retrospective cohort study was conducted to compare the clinical outcomes of patients receiving telemedicine compared to routine care at the outpatient clinics of the Medicine department from April 2020 to November 2021, including an original cohort (routine care: n = 54,032; telemedicine: n = 16,388) and a propensity score-matched cohort (n = 16,246 per group). Baseline and clinical characteristics, rates of unplanned visits and mortality outcomes were analyzed. Time prior to unplanned visits was calculated, and multivariate analysis was performed to identify predictors of unplanned visits.

**Findings:**

In the original cohort, the telemedicine group demonstrated a significantly higher incidence of unplanned outpatient visits (19.7% vs. 18.5%, p < 0.001). Conversely, in the matched cohort, the telemedicine group showed a lower rate of unplanned outpatient visits (19.4% vs. 59.3%, p < 0.001). Multivariate analysis confirmed that telemedicine was independently associated with a reduced risk of unplanned visits (adjusted HR: 0.69, 95% CI: 0.65 – 0.74, p < 0.001). However, other predictors with an increased incidence of unplanned visits included female patients, patients with chronic kidney disease stage 3 to 5, cancer and serum albumin levels below 4 g/dL. Additionally, telemedicine is associated with significantly lower mortality rates compared to routine care.

**Interpretation:**

Telephone-based telemedicine can be a viable alternative to routine care, offering comparable or improved outcomes in terms of a reduced rate of unplanned visits and lower mortality rates, particularly when combined with appropriate patient selection and monitoring strategies.

**Supplementary Information:**

The online version contains supplementary material available at 10.1186/s12877-025-06588-z.

## Introduction

Increasingly, telemedicine has become an important tool for delivering healthcare services in modern digital society. As defined by the World Health Organization, telemedicine refers to the use of information and communication technologies to deliver healthcare services when distance is a critical factor [1]. Telemedicine encompasses a variety of medical services including consultations, diagnosis, treatment and monitoring. The service could be delivered through various communication tools such as text messaging, telephone calls, mobile applications and videoconference, depending on the availability of the tools among providers and clients. The goal of telemedicine is to improve access to healthcare, which might also increase patient engagement and reduce healthcare costs [1]. Arranging telemedicine as a regular service might also help to tackle hospital overcrowding [2]. 

During the surge period of the COVID-19 pandemic, lockdown and social distancing policies were announced worldwide to control the spread of the virus. However, patients with chronic illnesses continued to require access to healthcare services. Therefore, telemedicine played a crucial role in delivering medical services that complied with infection control policy by reducing the chances of COVID-19 exposure for both patients and healthcare providers [3, 4]. Prior to the COVID-19 pandemic, the provision of telemedicine in most low-to-middle income countries was limited. There are still some obstacles to its widespread adoption mainly due to the lack of awareness among patients and healthcare providers, the limitation of technology access in some areas of the country and the lack of technological skills and familiarity with the relevant technology among older people [5].

Telephone-based telemedicine is a mode of delivering medical care remotely through telephone calls [6, 7]. One of the primary benefits of telephone-based telemedicine is its accessibility. It could potentially be delivered with limited technology and widely implemented in a short amount of time. However, it is not suitable for every medical condition with limitations in clinical assessment through visual evaluation only. During the COVID-19 pandemic crisis, there was an urgent need to provide care for a large number of non-COVID patients who required continuing medical care.

In Thailand, telemedicine was first implemented in most hospitals during the pandemic with various models for service delivery. Siriraj hospital, the largest medical school in Thailand has also developed telemedicine workflows to serve non-COVID patients. Telephone-based telemedicine was also selected as one of the options considering the existing limitations of the technological knowledge of a significant proportion of patients and the availability of devices. At the Department of Medicine, a proactive model to reach out to patients using telephone-based telemedicine was initiated under the “Telephone to Treatment (2T) SAVE-COVID” project. This project was a collaboration among multidisciplinary teams including physicians, nurses, pharmacists and all support divisions in the hospital.

Although telemedicine has shown great promise as a viable alternative to traditional healthcare delivery models, there are concerns for patient safety, particularly with any service where patients are not visible to the clinicians. Nevertheless, if telephone-based telemedicine could be proven to be a safe model in providing healthcare services, it is more likely that telemedicine will become an even more integral part of the healthcare system, providing patients with greater access to care and improving health outcomes. This study aimed to investigate the clinical outcomes of patients after receiving care via telephone-based telemedicine.

## Methods

### Policy formulation and implementation for telemedicine service

Prior to the implementation of the workflow, there were meetings in the Department of Medicine to set a clinical flow that was considered appropriate for a proactive outreach approach in the 2T-SAVE COVID project. It was decided to provide telemedicine for patients with stable chronic diseases requiring ongoing medical care. There were subsequently several meetings arranged among the disciplines in the hospital to set the workflow and documentation required to ensure the quality of care and allay safety concerns. 

For the telemedicine in 2T workflow, physicians would take the patient’s history and provide consultation through telephone or video calls and documented this in their medical records. If the patients reported any concerning symptoms, physicians would make a recommendation to go to the nearest hospital or Siriraj hospital at the patients’ convenience. Nurses would check the medical records for any future appointment and prepare to deliver the documents needed. Pharmacists would prepare the medications, provide additional consultation for high-risk medications, and deliver the medication package by post or fast-track self-pickup, depending upon convenience for the patients. Patients with malignancy requiring ongoing active treatments would be excluded from the telemedicine workflow and would have to come for onsite treatment as originally scheduled. In the video mode, there were additional technical requirements, including patients having a smartphone and setting up the application. During the pandemic surge, the workflow was arranged and the recommendation for telemedicine was announced by the head of the department. However, the implementation of the telemedicine workflow was not compulsory for all physicians in the department. 

### Study design and data collection

This study was a retrospective cohort exporting the patients’ characteristics from the hospital electronic database and prospectively identifying clinical outcomes using data linkage. All patients who had appointments at the outpatient clinics under the Department of Medicine at Siriraj hospital from April 2020 to November 2021 were identified in the hospital database. New patients, defined as those who had no outpatient visits from April 2019 to April 2020, were excluded. Patients who were not suitable for telemedicine workflow such as patients in hematology and oncology clinics, were excluded. The remaining population was divided into the routine care group and the telemedicine group based on the service setting in which the patients received care. 

**Fig. 1:**
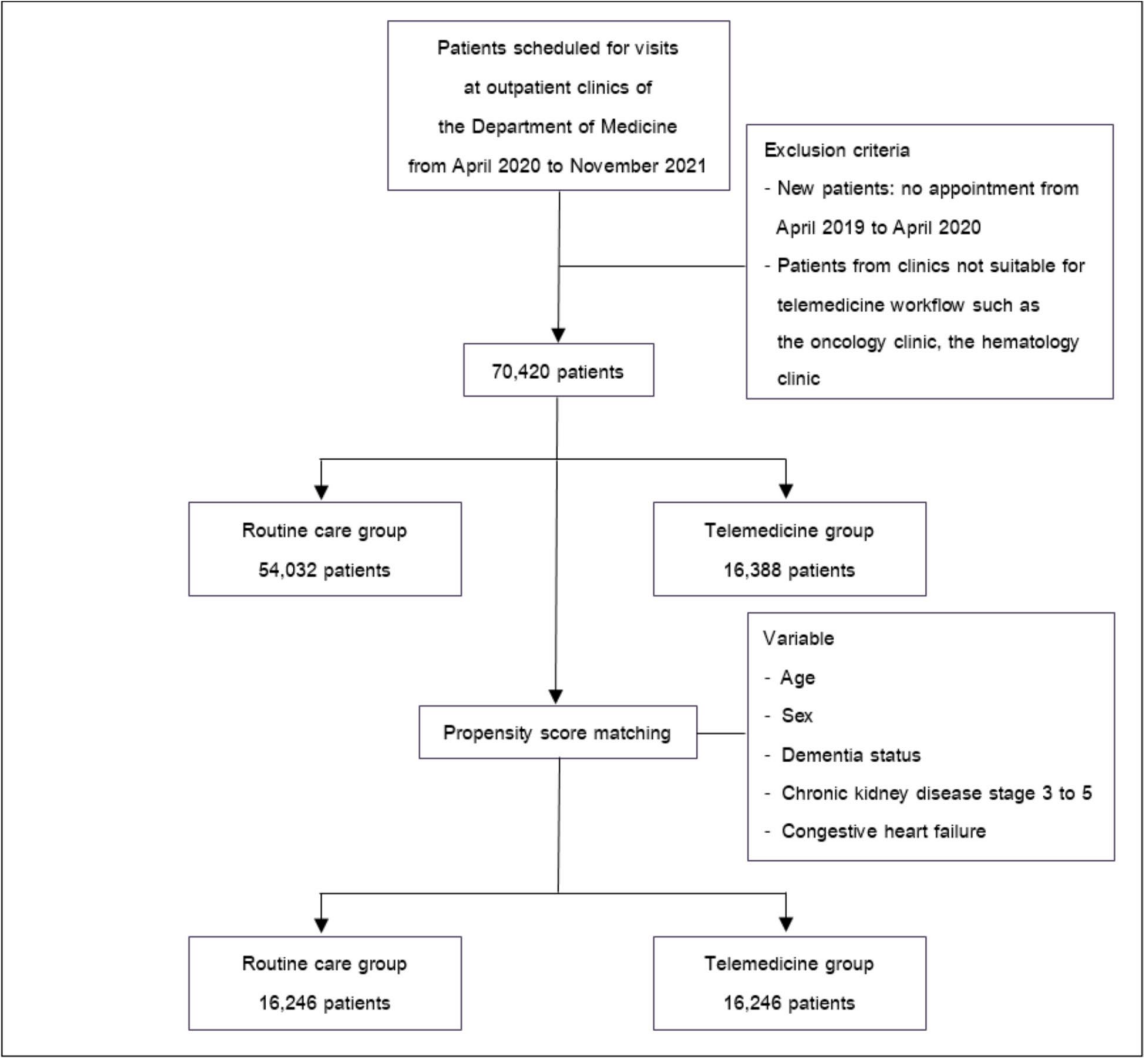
Flow of data retrieved from hospital database

The data was systematically sampled to ensure the validity and reliability of the retrieved data. Baseline characteristics including age, sex, comorbidities, the number of medications and laboratory investigations were identified. All-cause mortality was obtained from the Siriraj database and the civil registration. This study protocol was approved by the Siriraj Institutional Review Board (Fig. 1).

### Outcomes of interest

#### Primary outcome

The primary outcome of the study was the rate of unplanned visits, which were defined as hospital visits occurring outside of the scheduled appointment date. In the telemedicine group, unplanned visits were required to occur after using the telemedicine service. Unplanned visits included both inpatient and outpatient visits.

#### Secondary outcomes

The secondary outcomes of the study included the factors associated with unplanned visits. Data on the causes of unplanned visits and mortality were obtained from the Siriraj hospital database and the civil registration. The related medical conditions were identified using ICD-10 records in the Siriraj hospital database.

### Statistical analysis

Baseline characteristics were analyzed by using descriptive statistics. Categorical variables were analyzed by using Chi-square test or Fisher’s exact test as appropriate. All continuous data was tested for normality. The independent sample t-test and Mann–Whitney U test were used to compare continuous variables based on the data distribution. 

Propensity score matching was performed with a ratio of 1:1 and the match tolerance was set at 0.0001. The following variables expected to affect treatment outcomes were selected: age, sex, dementia status, chronic kidney disease stage 3 to 5 and congestive heart failure. Standardized differences were used to diagnose the baseline balance after matching. A standardized difference of less than 0.1 represented an adequate variable balance between groups. The propensity score was calculated using a logistic regression model. The adequacy of the propensity score matching models was evaluated with c-statistics in receiver operating characteristic analysis. After matching, the clinical outcomes of the matched pairs were compared.

Multivariate analyses were performed to evaluate the associations between potential factors and the primary outcome. The multivariate analysis was conducted to compare unplanned visits in both groups. The variables adjusted included mode of care, sex, underlying diseases and serum albumin. The Kaplan–Meier survival curve illustrates the cumulative survival rates over 24 months. A *p*-value < 0.05 was considered statistically significant. The statistical analysis was performed by using SPSS for Windows version 29 software.

## Results

### Demographic and Clinical Characteristics. (Table 1)

**Table 1:** Demographic and clinical characteristics of included subjects

	**Original cohort**			**Matched cohort**			
	**Routine care** **(n = 54,032)**	**Telemedicine** **(n = 16,388)**	**p-value**	**Routine care** **(n = 16,246)**	**Telemedicine** **(n = 16,246)**	**p-value**	**SMD**
Age (mean ± SD)	62.03 ± 15.30	63.73 ± 15.36	< 0.001	63.78 ± 15.31	63.69 ± 15.35	0.590	0.006
Age < 60, n (%)	20,516 (38.0)	5,498 (33.5)	< 0.001	5,424 (33.4)	5,464 (33.5)	0.891	
Age 60–79, n (%)	27,210 (50.4)	8,452 (51.6)		8,409 (51.8)	8,386 (51.6)		
Age ≥ 80, n (%)	6,306 (11.7)	2,438 (14.9)		2,412 (14.8)	2,396 (14.7)		
Sex							
Female, n (%)	34,792 (64.4)	10,078 (61.5)	< 0.001	10,036 (61.8)	9,976 (61.4)	0.494	0.008
Address			< 0.001			< 0.001	0.22
Bangkok, n (%)	28,909 (53.5)	8,122 (49.6)		9,769 (60.1)	8,033 (49.4)		
Others, n (%)	25,123 (46.5)	8,266 (50.4)		6,477 (39.9)	8,213 (50.6)		
Number of medications(median (min, max)), n (%)	7 (0, 19)	9 (0, 16)	< 0.001	9 (0, 19)	9 (0, 16)	< 0.001	0.002
0	3,533 (7.1)	697 (4.4)		753 (4.9)	697 (4.4)		
1–4	11,150 (22.3)	2,724 (17.2)		2,701 (17.4)	2,713 (17.3)		
5–10	19,411 (38.8)	6,343 (40.1)		5,683 (36.7)	6,288 (40.1)		
> 10	15,932 (31.8)	6,040 (38.2)		6,348 (41.0)	5,966 (38.1)		
Comorbidity, n (%)							
Hypertension	34,373 (63.6)	11,613 (70.9)	< 0.001	10,995 (67.7)	11,509 (70.8)	< 0.001	0.06
Diabetes mellitus	19,364 (35.8)	6,091 (37.2)	0.002	6,443 (39.7)	6,036 (37.2)	< 0.001	0.05
Myocardial infarction	1,314 (2.4)	855 (5.2)	< 0.001	537 (3.3)	847 (5.2)	< 0.001	0.09
Congestive heart failure	3,409 (6.3)	1,732 (10.6)	< 0.001	1,639 (10.1)	1,714 (10.6)	0.171	0.01
CVA	6,402 (11.8)	2,624 (16.0)	< 0.001	2,369 (14.6)	2,599 (16.0)	< 0.001	0.03
CKD stage ≥ 3	10,500 (19.4)	3,436 (21.0)	< 0.001	3,376 (20.8)	3,409 (21.0)	0.653	0.004
Dementia	1,975 (3.7)	1,064 (6.5)	< 0.001	980 (6.0)	1,050 (6.5)	0.109	0.02
CCI (median (min,max)), n (%)	1 (0, 18)	1 (0, 14)	< 0.001	1 (0, 17)	1 (0, 14)	< 0.001	0.17
CCI < 5	49,337 (91.3)	15,537 (94.8)	< 0.001	14,668 (90.3)	15,405 (94.8)	< 0.001	
CCI ≥ 5	4,695 (8.7)	851 (5.2)		1,578 (9.7)	841 (5.2)		
Laboratory investigation (mean ± SD)							
Hematocrit; Hct (%)	38.25 ± 5.08	38.52 ± 4.84	< 0.001	38.07 ± 5.20	38.52 ± 4.84	< 0.001	0.09
Serum creatinine; Cr (mg/dL)	1.04 ± 1.08	1.01 ± 0.96	< 0.003	1.06 ± 1.08	1.01 ± 0.96	< 0.001	< 0.1
Serum albumin (g/dL)	4.15 ± 0.46	4.16 ± 0.44	0.110	4.10 ± 0.48	4.16 ± 0.44	< 0.001	0.13
Albumin < 4, n (%)	5,255 (25.2)	1,683 (24.4)		2,032 (29.8)	1,669 (24.4)		
Albumin ≥ 4, n (%)	15,632 (74.8)	5,227 (75.6)		4,778 (70.2)	5,180 (75.6)		
HbA1C (mg%)	6.37 ± 1.16	6.26 ± 1.02	< 0.001	6.42 ± 1.20	6.26 ± 1.02	< 0.001	0.14

*CCI* Charlson Comorbidity Index, *SMD* Standardized mean differences

The adjusted covariates included age, sex, dementia status, chronic kidney disease stage 3–5 and congestive heart failure

In the original cohort of 54,032 routine care patients and 16,388 telemedicine patients, there were notable differences in demographics and clinical profiles. Telemedicine patients were slightly older, with a mean age of 63.73 years compared to 62.03 years in routine care patients (p < 0.001). The proportion of female patients was lower in the telemedicine group (61.5% vs. 64.4%, p < 0.001). Geographic distribution differed, with fewer telemedicine patients residing in Bangkok (49.6% vs. 53.5%, p < 0.001). Additionally, telemedicine patients had a higher median number of medications (9 vs. 7, p < 0.001), and a larger proportion were on more than 10 medications (38.2% vs. 31.8%).

Comorbidities were more prevalent in the telemedicine group, including hypertension (70.9% vs. 63.6%, p < 0.001) and diabetes mellitus (37.2% vs. 35.8%, p = 0.002). However, despite having a higher chronic disease burden, fewer telemedicine patients had a high Charlson Comorbidity Index (CCI ≥ 5) compared to routine care patients (5.2% vs. 8.7%, p < 0.001).

After propensity score matching, which balanced key baseline characteristics, the demographic and clinical profiles became comparable. Age and sex distributions were similar, with a mean age of approximately 63.7 years and 61.4% of patients being female in the telemedicine group compared to 61.8% in routine care. Differences in geographic distribution persisted, with fewer telemedicine patients residing in Bangkok (49.4% vs. 60.1%, p < 0.001). The telemedicine group continued to have a lower median number of medications with 38.1% of patients using more than 10 medications compared to 41.0% in routine care.

### The rate of unplanned visits and the rate of mortality. (Table 2)

**Table 2:** The rates of unplanned visits and the rate of mortality compared between the routine care group and the telemedicine group

	**Original cohort**			**Matched cohort**		
	**Routine care** **(n = 54,032)**	**Telemedicine** **(n = 16,388)**	**p-value**	**Routine care** **(n = 16,246)**	**Telemedicine** **(n = 16,246)**	**p-value**
**Unplanned visits, n (%)**						
OPD	9,990 (18.5)	3,227 (19.7)	< 0.001	9,630 (59.3)	3,188 (19.4)	< 0.001
IPD	632 (1.2)	46 (0.3)	< 0.001	605 (3.7)	46 (0.3)	< 0.001
**In hospital mortality (IPD), n (%)**	206/632 (32.6)	15/46 (32.6)	1.000	193/605 (31.9)	15/46 (32.6)	0.921
All-cause mortality*, from last visit						
at 1 month	144 (0.3)	54 (0.3)	0.182	39 (0.2)	54 (0.3)	0.121
at 3 months	486 (0.9)	139 (0.8)	0.540	148 (0.9)	139 (0.9)	0.594
at 6 months	1,043 (1.9)	292 (1.8)	0.222	351 (2.2)	290 (1.8)	0.015
at 12 months	2,058 (3.8)	578 (3.5)	0.096	803 (4.9)	575 (3.5)	< 0.001

^*^The all-cause mortality data retrieved from the civil registration

In the original cohort, unplanned outpatient visits (OPD) occurred at a slightly higher rate in the telemedicine group (19.7%) compared to the routine care group (18.5%), with a statistically significant difference (*p* < 0.001). However, unplanned inpatient visits (IPD) were markedly lower in the telemedicine group (0.3%) compared to the routine care group (1.2%, *p* < 0.001). Mortality outcomes favored the telemedicine group, with lower in-hospital mortality (18.7% vs. 32.6%, *p* = 0.002). All-cause mortality rates at 1, 3, 6 and 12 months also demonstrated lower figures in the telemedicine group, such as a 12-month mortality rate of 3.5% compared to 3.8% in the routine care group, though the difference was not statistically significant (*p* = 0.096).

In the matched cohort, unplanned outpatient visits were significantly lower in the telemedicine group (19.4%) compared to the routine care group (59.3%, p < 0.001). Unplanned inpatient visits followed the same trend, with only 0.3% in the telemedicine group versus 3.7% in the routine care group (p < 0.001). In-hospital mortality was higher in the telemedicine group (32.6%) compared to the routine care group without statistically significant (31.9%, p = 0.921). The all-cause mortality at 12 months was significantly lower in the telemedicine group (3.5%) compared to the routine care group (4.9%, *p* < 0.001).

### The clinical factors associated with unplanned visits. (Table 3)

**Table 3:** Clinical factors associated with unplanned visits of the outpatient setting

Variables	Original cohort				Matched cohort			
	**Univariate analysis**		**Multivariate analysis**		**Univariate analysis**		**Multivariate analysis**	
	**Crude HR (95%CI)**	**p-value**	**Adjust HR (95%CI)**	**p-value**	**Crude HR (95%CI)**	**p-value**	**Adjust HR (95%CI)**	**p-** **value**
Mode of care								
Routine care	Ref		Ref		Ref		Ref	
Telemedicine	2.71 (2.59–2.83)	< 0.001	2.33 (2.19–2.48)	< 0.001	0.70 (0.67–0.73)	< 0.001	0.69 (0.65–0.74)	< 0.001
Sex								
Female	Ref		Ref					
Male	1.08 (1.04–1.12)	< 0.001	1.06 (1.00–1.12)	0.028				
Comorbidity								
Hypertension	1.24 (1.19–1.28)	< 0.001	0.99 (0.93–1.06)	0.861				
Diabetes mellitus	1.19 (1.15–1.23)	< 0.001	1.02 (0.96–1.07)	0.600	1.06 (1.03–1.10)	< 0.001	1.03 (0.98–1.09)	0.184
Myocardial infarction	1.55 (1.42–1.69)	< 0.001	1.08 (0.95–1.22)	0.257				
Congestive heart failure	1.83 (1.73–1.93)	< 0.001	1.39 (1.28–1.52)	< 0.001				
CKD stage ≥ 3	1.03 (0.99–1.07)	0.116	0.99 (0.93–1.05)	0.717	1.25 (1.19–1.30)	< 0.001	1.23 (1.16–1.30)	< 0.001
Cancer	1.15 (1.09–1.19)	< 0.001	1.15 (1.08–1.21)	< 0.001	1.27 (1.22–1.33)	< 0.001	1.17 (1.11–1.24)	< 0.001
Dementia	1.11 (1.04–1.19)	0.001	1.04 (0.96–1.14)	0.326	1.15 (1.07–1.23)	< 0.001	1.02 (0.93–1.12)	0.662
Serum albumin								
Albumin ≥ 4	Ref		Ref		Ref		Ref	
Albumin < 4	1.32 (1.25–1.39)	< 0.001	1.09 (1.03–1.16)	0.002	1.17 (1.11–1.25)	< 0.001	1.10 (1.04–1.17)	0.001

The number of unplanned visits in the IPD setting is low; therefore, the analysis focuses solely on the OPD setting. In the original cohort, univariate and multivariate analyses revealed that telemedicine was significantly associated with a lower risk of unplanned visits compared to routine care. The adjusted hazard ratio (HR) for telemedicine was 2.33 (95% CI: 2.19–2.48, p < 0.001) in the multivariate analysis. Male patients showed a slight increase in risk (adjusted HR: 1.06, 95% CI: 1.00–1.12, p = 0.028). Comorbidities such as congestive heart failure and cancer were also significant factors, with adjusted HRs of 1.39 (95% CI: 1.28–1.52, p < 0.001) and 1.15 (95% CI: 1.08–1.21, p < 0.001), respectively. Lower serum albumin levels (< 4 g/dL) were associated with an increased risk of unplanned visits (adjusted HR: 1.09, 95% CI: 1.03–1.16, p = 0.002).

In the matched cohort, telemedicine consistently demonstrated a protective effect against unplanned visits, with an adjusted HR of 0.69 (95% CI: 0.65–0.74, p < 0.001) in the multivariate analysis. Among comorbidities, chronic kidney disease (CKD) stage 3 to 5, cancer and lower serum albumin levels were significant predictors. CKD stage 3 to 5 had an adjusted HR of 1.23 (95% CI: 1.16–1.30, p < 0.001), cancer had an adjusted HR of 1.17 (95% CI: 1.11–1.24, p < 0.001), and serum albumin < 4 g/dL had an adjusted HR of 1.10 (95% CI: 1.04–1.17, p = 0.001).

**Fig. 2:**
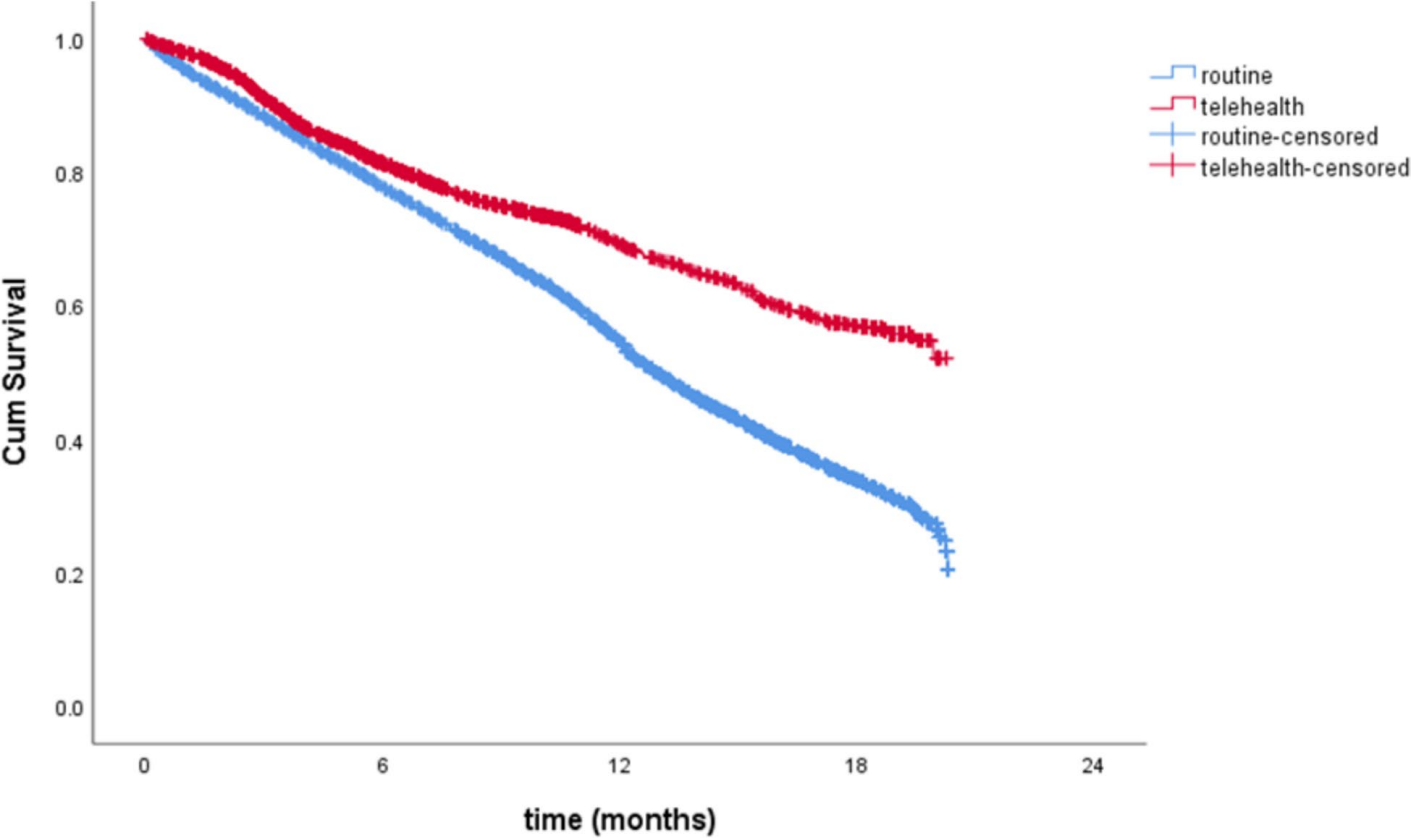
Cumulative survival curve comparing routine care and telemedicine group. This Kaplan–Meier survival curve illustrates the cumulative survival rates over 24 months for patients in the routine care group (blue line) and the telemedicine group (red line)

In the routine care group, there were 10,235 unplanned visits over a follow-up period of 185,148.93 person-months. This corresponds to a crude rate of 5.53 unplanned visits per 100 person-months, with a 95% confidence interval (CI) of 5.42–5.64. The telemedicine group recorded 3,234 unplanned visits over 99,803.86 person-months of follow-up. The crude rate of unplanned visits in this group was significantly lower at 3.24 visits per 100 person-months (95% CI: 3.13–3.35) (Table 4, Fig. 2).

**Table 4:** Comparison of unplanned visit rates between routine care and telemedicine groups (crude rates per person-month)

	**Routine care group**			**Telemedicine group**		
	**Event**	**Person-month** **of follow-up**	**Crude rate per** **person-month (95%CI)**	**Event**	**Person-month** **of follow-up**	**Crude rate per** **person-month (95%CI)**
Unplanned visit	10,235	185,148.93	5.53 (5.42–5.64)	3,234	99,803.86	3.24 (3.13–3.35)

## Discussion

This study provides valuable insights into the safety outcomes of patients receiving a telephone-based telemedicine service at outpatient clinics. The findings have indicated that patients who received care through a telemedicine service had a lower likelihood of having unplanned visits. Additionally, these outcomes enhance the importance of understanding telemedicine’s dual role in facilitating the selection of appropriate patients and effectively managing the medication dispensing system.

The outcome of the study after matching aligns with several randomized trials that have reported beneficial effects of telemedicine [8–12]. However, the results of the study contradicted a large cohort study demonstrating that patients who received telemedicine were more likely than their matched controls to experience emergency admissions or death [13]. In addition, two systematic reviews also failed to demonstrate a clear reduction in unplanned visits for patients receiving telemedicine services. It is worth noting that the methodological quality of the included studies in those reviews were at high risk of bias, and significant variations in clinical settings and telemedicine services were observed [14–16].

The positive outcomes observed in patients receiving care through telemedicine in this study can be attributed to several critical factors. First, the well-defined workflow developed in collaboration with the teams involved ensured the systematic and efficient delivery of telemedicine services. Second, the careful pre-appointment selection of patients by physicians, where telemedicine was deemed suitable, played a significant role. Physicians also retained the discretion to reassess the appropriateness of telemedicine during consultations, ensuring that only suitable cases continued with this mode of care [17]. Therefore, the high-risk patients who were recently discharged from the emergency department or the acute care setting were not appropriate for telemedicine services because these patients with a higher risk of revisits and readmissions were excluded from telemedicine services [18]. It is essential to emphasize that the study deliberately selected patients suitable for remote care, excluding high-risk individuals. This approach reflects real-world clinical practices that prioritize patient safety and treatment efficacy. By focusing on stable patients, the study provides a practical representation of how telemedicine can be effectively integrated into routine healthcare delivery [18–20].

Another key aspect of telemedicine in the study was the comprehensive approach to medication dispensing. The availability of medication delivery options, either via mail or direct pick-up, significantly enhanced patient adherence to treatment protocols [21]. This feature ensured that patients could reliably access their medications, thus promoting continuity of care.

Additionally, the timing of telemedicine implementation during the pandemic greatly amplified its benefits. During a period when access to public and healthcare facilities was severely limited, telemedicine offered a proactive solution for providing essential medical care. By facilitating consultations, providing medical advice, and ensuring medication delivery to vulnerable patients, telemedicine bridged the gap in healthcare access. In contrast, patients receiving usual care may have faced significant barriers to obtaining even basic healthcare services during this time.

Moreover, the findings revealed that unplanned visits occurred approximately 8 months after the first appointment in the routine care group. In contrast, unplanned visits occurred after only 4 months in the telemedicine group (Supplement; Table S1). This suggests that difficulties in traveling to the hospital for follow-up appointments may act as a barrier, resulting in a higher frequency of unplanned visits in the routine care group.

Telemedicine serves as a time-efficient healthcare delivery model. The convenience of virtual consultations reduced barriers such as travel [22], thereby improving adherence to follow-up appointments. This enhanced adherence likely contributed to better health outcomes and increased the overall effectiveness of telemedicine services. However, it may be beneficial to schedule follow-up appointments earlier, potentially before the 4-month mark, to further optimize care and prevent unplanned visits.

The study also examines the safety and effectiveness of a telephone-based telemedicine service. It highlights the benefits of a well-structured support process and clinical workflow for patient selection, demonstrating that advanced technology or video-based devices are not always necessary for effective care [17, 19, 20, 23]. The safety and efficacy of the service were particularly evident in vulnerable populations, such as older patients with dementia status, within a pre-specified subgroup. By delivering care through primary caregivers at home, the service successfully reduced unplanned visits for older individuals with dementia status. This outcome suggests that such an approach could be highly applicable in resource-limited settings where the implementation of advanced telemedicine systems is not feasible.

In Thailand, despite its status as a high middle-income country, many areas, even within the capital city, continue to face challenges related to poor-quality telecommunication networks and limited access to smartphones, particularly among the older population [24–27]. Unlike video-based telemedicine, which requires advanced telecommunication infrastructure, telephone-based services rely on basic mobile phone technology, which is widely available to patients. This enables healthcare providers to deliver essential care through simple phone calls. Therefore, this study provides evidence that telephone-based telemedicine can be a viable and safe option in resource-constrained settings, provided that healthcare providers carefully select appropriate patients and establish a robust support system for the service.

Although the service was initially implemented during the COVID-19 pandemic, its benefits extend far beyond infection control [3, 5, 6]. Telemedicine reduced the need for patient transportation, enhanced convenience, and ensured continuity of care for vulnerable populations. These advantages remain relevant in post-pandemic healthcare delivery, particularly in addressing outpatient department overcrowding and improving access for immobile patients [4, 6, 7].

However, the structured workflow and the careful patient selection in this study were critical to its success. Physicians ensured that telemedicine was reserved for patients who could be safely managed remotely, emphasizing its applicability in real-world settings [16, 17, 21, 23]. Optimizing telemedicine workflows to balance service utilization with resource allocation should be concerned in the future. Long-term evaluations of patient satisfaction, cost-effectiveness and the integration of telemedicine with traditional care models will further refine its application and impact [14, 15, 22].

### Strengths and limitations

The strength of this study lies in its large sample size, enabling robust analysis and increasing generalizability. The study was conducted during the period of the COVID-19 outbreak in Thailand, rendering it a pertinent and practical model of telemedicine during the pandemic. Additionally, the study examined clinical outcomes including all-cause mortality associated with having a telemedicine service, providing valuable insights into its effectiveness.

However, there were limitations to this study. Firstly, the study design was a retrospective cohort study; some crucial information, such as that regarding interventions provided during the consultations, was not collected, and limited the opportunity to demonstrate the genuinely beneficial interventions delivered. The retrospective design of this study may introduce biases and limitations inherent to observational research. These include potential selection bias, unmeasured confounding variables, and reliance on the accuracy of recorded data, which can impact the generalizability and robustness of the findings. Additionally, the presence of substantial missing data further complicates the analysis, potentially reducing the reliability and validity of the results (Supplement; Table S2). Furthermore, the study relied solely on the Siriraj hospital database and did not include data on unplanned visits from other hospitals, potentially resulting in an underestimation of unplanned visits. However, the attempts to collect data on all-cause mortality and proven benefits would minimize this potential bias. 

## Conclusion

This study highlights the critical role of telemedicine in reducing hospital visits, lowering mortality and improving long-term patient outcomes. The consistent results in both the original and matched cohorts underscore effectiveness of telemedicine in providing timely and accessible care, particularly in resource-limited settings.

## Supplementary Information

Supplementary Material 1.

Supplementary Material 2. Table S1: The time prior to unplanned visits. Table S2: Missing data.

## Data Availability

Data is provided within the supplementary information files.

## Abbreviations

CCI: Charlson Comorbidity Index
CI: Confidence interval
CKD: Chronic kidney disease
COVID-19: Coronavirus disease 2019
Cr: Creatinine
Cum survival: Cumulative survival
CVA: Cerebrovascular accident
ESRD: End-stage renal disease
Etc.: Et cetara
g/dL: Gram per deciliter
GI: Gastrointestinal
HbA1c: Hemoglobin A1C
Hct: Hematocrit
HN: Hospital numbers
HR: Hazard ratio
ICD-10: The International Classification of Diseases (ICD)-10
ICH: Intracranial hemorrhage
ID clinic: Infectious disease clinic
IPD: Inpatient department
LDL: Low-density lipoprotein
Med: Internal medicine clinics
mg/dL: Milligram per deciliter
N: Number
Non-Med: Clinics other than internal medicine clinics
OPD: Outpatient department
Ref: Reference
RR: Relative risk
SD: Standard deviation
SIRB: Siriraj Institutional Review Board
UGIB: Upper gastrointestinal bleeding
2T: Telephone to Treatment
