# Supplementary material for: The impact of telephone-based telemedicine on unplanned hospital visits and mortality risk during the COVID-19 pandemic: a study from a middle-income country
Source: BMC Geriatr. 2025 Dec 2;25:993. doi: 10.1186/s12877-025-06588-z (PMC12673738; doi:10.1186/s12877-025-06588-z)

**Supplementary**

| **Table S1: The time duration before unplanned visits.** | | | |
| --- | --- | --- | --- |
|  | **Routine care**  **group**  **(n = 11,784)** | **Telemedicine group**  **(n = 3,342)** | **p-value** |
| **Time to unplanned visits^¶^; months (mean ± SD)** | 3.3 ± 2.2 | 4.4 ± 3.6 | <0.001 |

¶ Time to unplanned visit defined as the duration from the last routine or telemedicine visit to the date of the unplanned visit.

| **Table S2: Secondary outcomes.** | | | |
| --- | --- | --- | --- |
|  | **Routine care**  **(n = 32,380)** | **Telemedicine**  **(n = 16,395)** | **p-value** |
| **Specific conditions, n (%)** |  |  |  |
| Pressure injury | 442 (1.4) | 245 (1.5) | 0.259 |
| Functional decline | 1038 (3.2) | 522 (3.2) | 0.897 |
| Malnutrition | 242 (0.8) | 135 (0.8) | 0.365 |
| Frailty | 21 (0.1) | 7 (0.0) | 0.334 |
| Joint stiffness | 621 (1.9) | 299 (1.8) | 0.470 |
| **Causes of OPD unplanned visit, %** | | | |
| GI problems (diarrhea, constipation, flatulent) | 22.0 % | | |
| Fall | 18.3 % | | |
| Covid-19 infection | 12.2 % | | |
| Local infection | 9.8 % | | |
| Urinary symptom (frequency, retention, etc.) | 6.1 % | | |
| Syncope | 3.7 % | | |
| Edema | 3.7 % | | |
| Oligoarthritis | 2.4 % | | |
| Vertigo/dizziness | 2.4 % | | |
| Hypertension problem | 2.4 % | | |
| Chest symptoms (dyspnea, pain) | 2.4 % | | |
| Bedsore problem | 1.2 % | | |
| Hypoglycemia | 1.2 % | | |
| Seizure | 1.2 % | | |
| Others | 11.0 % | | |
| **Causes of IPD unplanned visit, %** | | | |
| Covid-19 infection | 18.8 % | | |
| Urinary tract infection | 13.7 % | | |
| Fall | 8.6 % | | |
| Pneumonia | 7.7 % | | |
| Sepsis | 6.8 % | | |
| Chest symptoms (dyspnea, pain) | 5.1 % | | |
| GI problems | 4.2 % | | |
| Infected wound/Pressure injury | 4.2 % | | |
| ICH | 3.3 % | | |
| Arrhythmia | 2.6 % | | |
| Cancer problem | 2.6 % | | |
| Congestive heart failure | 2.6 % | | |
| Delirium | 2.6 % | | |
| Renal disease (electrolyte imbalance, AKI) | 2.6 % | | |
| Gallstone/biliary disease | 2.6 % | | |
| Hyper/Hypoglycemia | 2.6 % | | |
| Stroke | 2.6 % | | |
| Hypertensive emergency/urgency | 1.7 % | | |
| LGIB | 1.7 % | | |
| Muscle and joint pain (oligoarthritis, etc.) | 1.7 % | | |
| Pleural disease | 1.7 % | | |

| **Table S3: Demographic and baseline characteristics of the patients in Geriatric clinic.** | | | |
| --- | --- | --- | --- |
|  | **Routine care group**  **(n = 927)** | **Telemedicine group**  **(n = 636)** | **p-value** |
| **Sex** |  |  | 0.053 |
| Female, n (%) | 650 (70.1) | 416 (65.4) |  |
| Male, n (%) | 277 (29.9) | 220 (34.6) |  |
| **Address** |  |  | 0.085 |
| Bangkok, n (%) | 655 (70.7) | 423 (66.5) |  |
| Others, n (%) | 272 (29.3) | 213 (33.5) |  |
| **Health coverage, n (%)** |  |  | 0.023* |
| Civil servant medical benefit scheme | 565 (60.9) | 358 (56.3) |  |
| Siriraj universal health coverage scheme | 95 (10.2) | 82 (12.9) |  |
| Non Siriraj universal health coverage scheme | 70 (7.6) | 69 (10.8) |  |
| Others | 196 (21.2) | 127 (20.0) |  |
| **Number of medications (median (min,max)), n (%)** | 13 (0,51) | 13 (0,58) | 0.640 |
| 0 | 12 (1.3) | 11 (1.7) |  |
| 1-4 | 56 (6.2) | 43 (6.8) |  |
| 5-10 | 247 (27.1) | 174 (27.7) |  |
| >10 | 595 (65.4) | 401 (63.8) |  |
| **Comorbidity, n (%)** |  |  |  |
| Hypertension | 803 (86.6) | 585 (92.0) | 0.001* |
| Diabetes mellitus | 420 (45.3) | 297 (46.7) | 0.606 |
| Myocardial infarction | 35 (3.8) | 27 (4.2) | 0.693 |
| Congestive heart failure | 90 (9.7) | 82 (12.9) | 0.058 |
| CVA | 266 (28.7) | 219 (34.4) | 0.017* |
| CKD stage ≥ 3 | 268 (28.9) | 216 (34.0) | 0.035* |
| ESRD | 95 (10.2) | 54 (8.5) | 0.256 |
| Dementia | 424 (45.7) | 374 (58.8) | <0.001* |
| Cancer | 171 (18.4) | 113 (17.8) | 0.739 |
| **CCI (median (min,max)), n (%)** | 2 (0,13) | 2 (0,11) | 0.044 |
| CCI < 5 | 84 (91.6) | 582 (91.5) |  |
| CCI ≥ 5 | 78 (8.4) | 54 (8.5) |  |
| **Laboratory investigation, (mean ± SD)** |  |  |  |
| Hematocrit; Hct (%) | 36.96 ± 4.80 | 36.94 ± 4.57 | 0.940 |
| Serum creatinine; Cr (mg/dL) | 1.04 ± 0.78 | 1.06 ± 0.76 | 0.554 |
| Serum albumin (g/dL) | 3.96 ± 0.47 | 3.93 ± 0.44 | 0.293 |
| HbA1C (mg%) | 6.29 ± 1.10 | 6.28 ± 1.18 | 0.843 |

| **Table S4: primary outcomes of the patients in Geriatric clinic.** | | | |
| --- | --- | --- | --- |
|  | **Routine care**  **(n = 927)** | **Telemedicine**  **(n = 636)** | **p-value** |
| **Unplanned visit, n (%)** | 484 (52.2) | 243 (38.2) | <0.001 |
| OPD | 467 (50.4) | 239 (37.6) | <0.001 |
| IPD | 17 (1.8) | 4 (0.6) | 0.042 |
| **In hospital mortality, n (%)** | 31 (3.3) | 15 (2.4) | <0.001 |

| **Table S5: Telephone interview** | | |
| --- | --- | --- |
|  | **Routine care group**  **(n = 411)** | **Telemedicine group**  **(n = 398)** |
| **All-cause Mortality, n (%)** | 50 (12.2) | 30 (7.5) |
| **COVID-19 infection, n (%)** | 89 (21.7) | 85 (21.4) |
| **Hospital of choice when illness, n (%)** |  |  |
| Siriraj hospital | 352 (85.6) | 337 (84.6) |
| Others | 59 (14.4) | 61 (15.4) |

| **Table S6: Factors associated with unplanned visits (exclude video appointments)** | | | | |
| --- | --- | --- | --- | --- |
|  | **Univariate analysis** | | **Multivariate analysis** | |
| **Variables** | **Crude HR (95%CI)** | **p-value** | **Adjust HR (95%CI)** | **p-value** |
| **Group** |  |  |  |  |
| Routine care | Ref |  | Ref |  |
| Telemedicine | 0.21 (0.20-0.22) | <0.001* | 0.22 (0.21-0.23) | <0.001* |
| **Age** |  |  |  |  |
| Age < 60 | Ref |  | Ref |  |
| Age ≥ 60 | 1.04 (1.02-1.07) | 0.003* | 0.93 (0.89-0.98) | 0.004* |
| **Comorbidity** |  |  |  |  |
| Hypertension | 1.16 (1.13-1.19) | <0.001* | 1.03 (0.97-1.08) | 0.389 |
| Diabetes mellitus | 1.30 (1.27-1.34) | <0.001* | 1.14 (1.09-1.19) | <0.001* |
| Congestive heart failure | 1.18 (1.13-1.24) | <0.001* | 1.19 (1.11-1.27) | <0.001* |
| CKD stage > 3 | 1.36 (1.32-1.40) | <0.001* | 1.26 (1.20-1.33) | <0.001* |
| Cancer | 1.15 (1.11-1.19) | <0.001* | 1.08 (1.03-1.13) | 0.001* |
| Dementia | 1.08 (1.02-1.14) | 0.004* | 0.99 (0.90-1.07) | 0.672 |
| **Serum albumin** | 0.84 (0.80-0.87) | <0.001* | 0.94 (0.90-0.98) | 0.015 |
| **Number of medications** |  |  |  |  |
| 0 | Ref |  | Ref |  |
| 1-4 | 1.04 (0.98-1.12) | 0.186 | 1.08 (0.95-1.23) | 0.221 |
| 5-10 | 1.13 (1.06-1.20) | <0.001* | 1.23 (1.08-1.39) | <0.001* |
| >10 | 1.38 (1.29-1.47) | <.0001* | 1.45 (1.28-1.64) | <0.001* |

**Figure S1: Survival analysis of unplanned visits for the entire cohort**
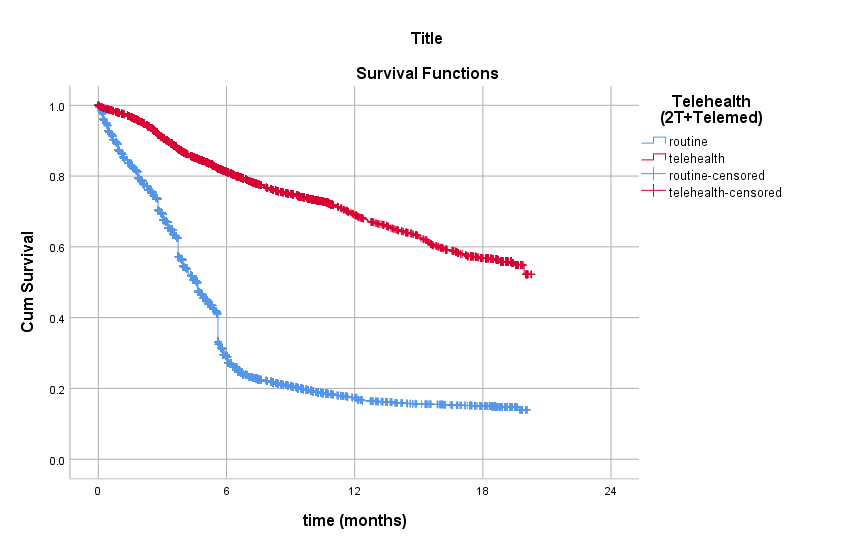

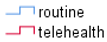


**Figure S2: Survival analysis of unplanned visits of the patients in Geriatric clinic.**


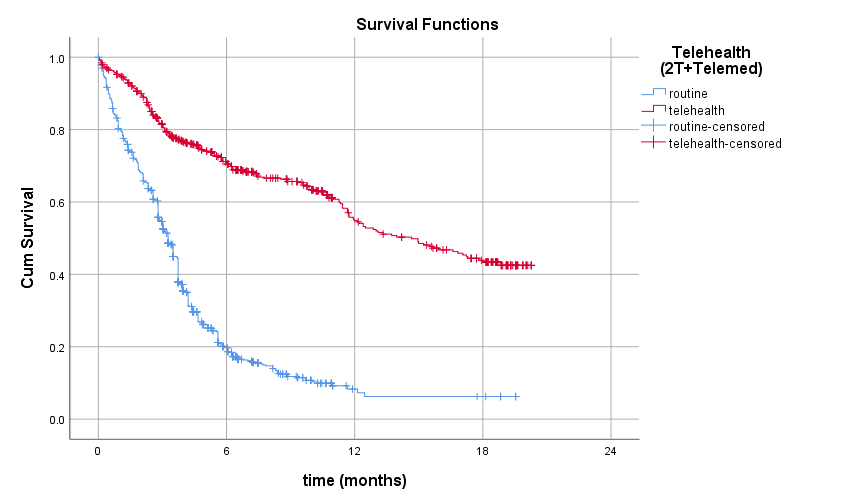

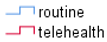

Supplement: Supplementary file 1 — Supplementary Material 1. [file 12877_2025_6588_MOESM1_ESM.docx]
