# Supplementary material for: The impact of telephone-based telemedicine on unplanned hospital visits and mortality risk during the COVID-19 pandemic: a study from a middle-income country
Source: BMC Geriatr. 2025 Dec 2;25:993. doi: 10.1186/s12877-025-06588-z (PMC12673738; doi:10.1186/s12877-025-06588-z)
Supplement: Supplementary file 2 — Supplementary Material 2. Table S1: The time prior to unplanned visits. Table S2: Missing data. [file 12877_2025_6588_MOESM2_ESM.docx]

**Supplement**

**Table S1: The time prior to unplanned visits.**

|  | **Original cohort** | | | **Matched cohort** | | |
| --- | --- | --- | --- | --- | --- | --- |
|  | **Routine care**  **(n=54,032)** | **Telemedicine**  **(n=16,388)** | **p-value** | **Routine care**  **(n=16,246)** | **Telemedicine**  **(n=16,246)** | **p-value** |
| **Time prior to unplanned visits** ¶**; months**  **(mean ± SD)** |  |  |  |  |  |  |
| OPD | 8.67 ± 5.06 | 4.38 ± 3.62 | <0.001 | 8.56 ± 5.03 | 5.00 ± 3.21 | <0.001 |
| IPD | 9.68 ± 6.79 | 5.01 ± 4.25 | <0.001 | 9.69 ± 6.84 | 5.01 ± 4.25 | <0.001 |

¶ Time prior to unplanned visits was defined as the duration from the first routine or telemedicine visits to the date of the unplanned visits.

**Table S2: Missing data.**

| **Variables** | **Original cohort (n=70,420)** | **Matched cohort (n=32,492)** |
| --- | --- | --- |
| **Age** | **-** | **-** |
| **Sex** | **-** | **-** |
| **Address** | **-** | **-** |
| **Number of medications** | 4,590 (6.5%) | 1,343 (4.1%) |
| **Comorbidity,** |  |  |
| Hypertension | - | - |
| Diabetes mellitus | - | - |
| Myocardial infarction | - | - |
| Congestive heart failure | - | - |
| CVA | - | - |
| CKD stage ≥3 | - | - |
| Dementia | - | - |
| **Charlson Comorbidity Index** | - | - |
| **Serum albumin** | 42,623 (60.5%) | 18,833 (58.0%) |
| **Laboratory investigation** |  |  |
| Hematocrit; Hct (%) | 28,780 (40.9%) | 12,090 (37.2%) |
| Serum creatinine; Cr (mg/dL) | 14,455 (20.5%) | 5,440 (16.7%) |
| HbA1C (mg%) | 37,759 (53.6%) | 16,451 (50.6%) |
